# Validity of TIMI Score for Predicting 14-Day Mortality of Non-ST Elevation Myocardial Infarction Patients

**DOI:** 10.7759/cureus.12518

**Published:** 2021-01-06

**Authors:** Dileep Kumar, Tahir Saghir, Maham Zahid, Arti Ashok, Mukesh Kumar, Arshad Ali Shah, Izza Shahid, Sajjad Ali, Ayema Haque, Musa Karim

**Affiliations:** 1 Cardiology, National Institute of Cardiovascular Diseases, Karachi, PAK; 2 Department of Medicine, Ziauddin University, Karachi, PAK; 3 Cardiology, Dow University of Health Sciences, Civil Hospital, Karachi, PAK; 4 Internal Medicine, Ziauddin Medical College, Karachi, PAK; 5 Internal Medicine, Dow University of Health Sciences, Civil Hospital, Karachi, PAK; 6 Statistics, National Institute of Cardiovascular Diseases, Karachi, PAK

**Keywords:** non-st elevation myocardial infarction, thrombolysis in myocardial infarction, 14-day mortality, prognosis, validation

## Abstract

Background

Accurate management of non-ST elevation myocardial infarction (NSTEMI) patients can be achieved by stratifying risks as early as possible on hospital admission. Previously, the Thrombolysis in Myocardial Infarction (TIMI) risk score has been validated and used on patients presenting with NSTEMI or unstable angina (UA) in developed countries. The aim of this study was to assess the validity of the TIMI risk score in patients presenting with NSTEMI in Pakistan.

Methods

This cross-sectional study was undertaken on 300 patients who were diagnosed with NSTEMI. Data were collected from medical records, the TIMI score was calculated, and 14-day outcome was recorded. The receiver operating characteristic (ROC) curve analysis was performed, and area under the curve (AUC) along with 95% confidence interval (CI) was reported. Univariate and multivariate logistic regression analysis was performed and odds ratio (OR) along with 95% CI was reported.

Results

This cross-sectional study was undertaken on 300 patients who were diagnosed with NSTEMI. Data were collected from medical records, the TIMI score was calculated, and 14-day outcome was recorded. Validity of TIMI score in predicting hospital mortality 14 days after the diagnosis of NSTEMI in a population in Pakistan was assessed by ROC curve and logistic regression analysis. The AUC of the TIMI score for predicting 14-day outcome was 0.788 [95% CI: 0.689-0.887], with optimal cutoff of ≥4 with sensitivity of 77.78%. On multivariate analysis, cardiac arrest at presentation and the TIMI risk score were found to be independent predictors of 14-day mortality with adjusted ORs of 136.49 [10.23-1821.27] and 2.67 [1.09-6.57], respectively.

Conclusions

The TIMI risk score is a useful and simple score for the stratification of patients with high risk of 14-day mortality with reasonably acceptable discriminating ability in patients with NSTEMI acute coronary syndrome.

## Introduction

Acute myocardial infarction (AMI) and unstable angina (UA) come under the broad umbrella of the term acute coronary syndrome (ACS), which accounts for nearly seven million deaths annually [1]. AMI is further classified according to ST segment deviation (ECG) which includes ST elevation myocardial infarction (STEMI) and non-ST elevation myocardial infarction (NSTEMI). Approximately 70% of ACS patients present with NSTEMI [2], for which the treatment options are less clear [3]. Patients presenting with NSTEMI are usually older and prone to increased risk of cardiovascular complications compared to patients with STEMI [4]. Despite improvement in therapeutic interventions in the recent decade, NSTEMI still accounts for high morbidity and mortality rates [5]. This makes it crucial to use adequate risk factor assessment to determine potentially fatal cardiac complications, which may enable physicians to provide suitable and timely therapeutic management to the most vulnerable patients, thereby reducing mortality rates [6-8].

The Thrombolysis in Myocardial Infarction (TIMI) risk score has been demonstrated to be an effective risk stratification tool for predicting in-hospital mortality or 14-day mortality among patients with NSTEMI. Multiple trials have validated the use of the TIMI risk score in NSTEMI patients [3,9-11], which is now one of the most widely used risk stratification model for patients presenting with NSTEMI. However, the development and validation of the TIMI risk score is primarily derived from developed countries, with limited data evaluating the effectiveness of the TIMI risk score in South Asian populations despite a higher cardiovascular disease (CVD) burden [12]. This is particularly important for lower-middle-income countries like Pakistan, which are increasingly affected by CVD epidemic and encompass different genetics, lifestyle, and healthcare services compared to developed countries. Although the predictive power of the TIMI risk score has previously been validated in patients with STEMI in Pakistan [13], limited data exist regarding the validity of this risk score in patients with NSTEMI. Therefore, we sought to evaluate the validity of the TIMI risk score for predicting 14-day mortality in NSTEMI patients presenting to the largest cardiac care center of the country in an effort to determine the prognostic significance of this risk score in this cohort.

## Materials and methods

This study was conducted at a tertiary care cardiac center in Karachi during September 2019 and December 2019. After obtaining approval from the Ethics Review Committee of the institution, adult patients of either gender who presented to the Emergency Department with NSTEMI were included in the study. Informed consent was obtained from all the patients regarding their participation in the study. Patients who refused to give consent or had a history of cardiovascular surgery(s) or intervention(s) were excluded. A structured proforma was designed consisting of demographic variables, history regarding comorbidities, baseline investigations, TIMI risk score at presentation, and outcome after 14 days of admission. All patients received the ACS protocol, and management for NSTEMI was uniform for all patients as per the institutional protocol and current guidelines. For all enrolled patients, an outpatient-based follow-up visit was planned after 14 days of indexed hospitalization and telephonic calls were made for those who missed the planned follow-up visit.

Collected data were analyzed using the Statistical Package for the Social Sciences (SPSS) version 21 (IBM, Chicago, USA). The receiver operating characteristic (ROC) curve analysis was performed taking the TIMI score as the test variable and 14-day mortality as the state variable, and the area under the curve (AUC) ± standard error (SE) along with its asymptotic 95% confidence interval (CI) were calculated. The ROC analysis was performed on R version 4.0.2 using the R-packages “ROCit,” “ROCR,” “dplyr,” and “ggplot2.” The optimal cutoff point was computed using the Youden Index method and patients were categorized into high- and low-risk groups based on the computed optimal cutoff point and its association to the 14-day outcome rates was assessed using the Chi-square test. The association between survival status after 14 days and demographic and clinical characteristics of the patients were evaluated by performing appropriate Chi-square test or Student’s t-test/Mann-Whitney U test. The univariate and multivariate binary logistic regression analysis were also performed to assess the strength of associations and odds ratios (ORs) and its 95% CIs were computed. Throughout the analysis p ≤ 0.05 was taken as the significance criteria.

## Results

A total of 300 patients with NSTEMI were included in this study. Of those, 76.7% were males while 23.3% were females. Overall, the mean age of the patients was 58.04 ± 10.71 years, with 21.7% (n = 65) of the patients aged 65 years and above. The overall mortality rate after 14 days of procedure was 6.0% (n = 18), which was significantly higher in patients with diabetes (10.3% [13/126] vs. 2.9% [5/174]; p = 0.007). Similarly, the 14-day mortality rate was significantly higher among patients with cardiac arrest (90.9% (10/11) vs. 2.8% (8/289); p < 0.001) compared to those without cardiac arrest. The 14-day mortality was found to be associated with Killip class and mean heart rate at presentation, which was significantly higher in patients who did not survive (84.33 ± 18.09 vs. 76.99 ± 13.94; p = 0.034). Additionally, the TIMI score was also associated with increased mortality, with a higher risk score observed in patients who did not survive compared to those who did (4.06 ± 0.73 vs. 3.12 ± 0.83; p < 0.001). The baseline clinical characteristics and TIMI score at presentation stratified by 14-day outcome are presented in Table 1.

**Table 1:** Clinical characteristics and TIMI score at presentation stratified by 14-day outcome CAD, coronary artery diseases; SBP, systolic blood pressure; TIMI, thrombolysis in myocardial infarction *significant

Characteristics	Total	Outcome at 14 days		p-value
		Survived	Expired	
N	300	282	18	-
Gender				
Male	230 (76.7%)	218 (77.3%)	12 (66.7%)	0.301
Female	70 (23.3%)	64 (22.7%)	6 (33.3%)	
Age (years)	58.04 ± 10.71	57.77 ± 10.41	62.33 ± 14.31	0.080
≤50 years	80 (26.7%)	75 (26.6%)	5 (27.8%)	0.144
51-65 years	155 (51.7%)	149 (52.8%)	6 (33.3%)	
> 65 years	65 (21.7%)	58 (20.6%)	7 (38.9%)	
Risk factors				
Hypertension	253 (84.3%)	236 (83.7%)	17 (94.4%)	0.224
Diabetes	126 (42%)	113 (40.1%)	13 (72.2%)	0.007*
Family history of CAD	33 (11%)	30 (10.6%)	3 (16.7%)	0.428
Smoking	82 (27.3%)	78 (27.7%)	4 (22.2%)	0.616
Obesity	27 (9%)	25 (8.9%)	2 (11.1%)	0.747
Dyslipidemia	46 (15.3%)	44 (15.6%)	2 (11.1%)	0.608
Sedentary lifestyle	93 (31%)	87 (30.9%)	6 (33.3%)	0.825
Killip class				
I	253 (84.3%)	250 (88.7%)	3 (16.7%)	<0.001*
II	39 (13%)	29 (10.3%)	10 (55.6%)	
III	6 (2%)	2 (0.7%)	4 (22.2%)	
IV	2 (0.7%)	1 (0.4%)	1 (5.6%)	
Cardiac arrest	11 (3.7%)	1 (0.4%)	10 (55.6%)	<0.001*
Heart rate (bpm)	77.43 ± 14.29	76.99 ± 13.94	84.33 ± 18.09	0.034*
SBP (mmHg)	120.99 ± 17.21	120.82 ± 16.97	123.72 ± 20.99	0.488
Serum creatinine (ng/dL)	1.45 ± 6.75	1.44 ± 6.96	1.61 ± 0.77	0.922
Troponin I (ng/dL)	7.12 ± 17.05	6.9 ± 17.31	10.48 ± 12.3	0.389
TIMI score	3.18 ± 0.85	3.12 ± 0.83	4.06 ± 0.73	<0.001*

**Figure 1:**
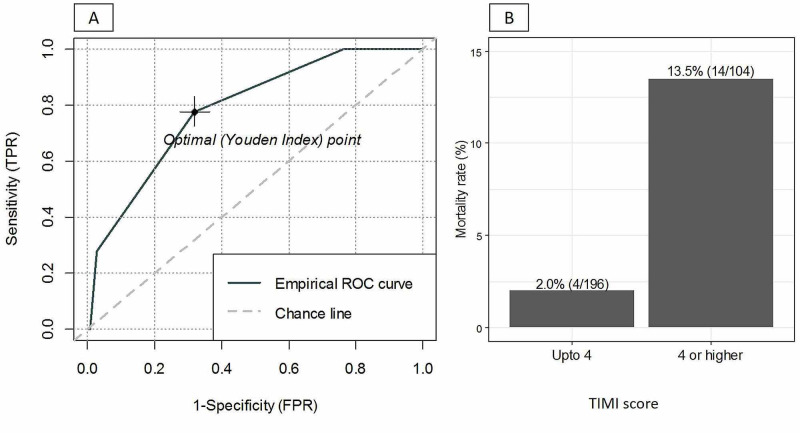
ROC curve (A) and mortality rate with respect to the TIMI score (B). ROC, receiver operating characteristic; TIMI, thrombolysis in myocardial infarction

The ROC curve and mortality rate with respect to the TIMI score are presented in Figure 1. The predictive value, AUC of TIMI score, for predicting 14-day outcome was found to be 0.788 (95% CI: 0.689-0.887), with optimal cutoff of ≥4 with sensitivity of 77.78% (95% CI: 52.36%-93.59%) and specificity of 68.09% (95% CI: 62.30%-73.49%). The mortality rate was significantly higher in patients with TIMI score of ≥4 compared to those with TIMI score <4, with 14-day mortality rates of 13.5% (14/104) versus 2.0% (4/196) (p < 0.001), respectively.

The univariate and multivariate logistic regression analysis for 14-day outcome are presented in Table 2. The univariate analysis revealed that heart rate (per 5 bpm increase), cardiac arrest, Killip class III or IV, and/or diabetes as comorbidity upon presentation are the key factors associated with significant increased risk of 14-day mortality with ORs of 1.16 [1.01-1.33], 351.25 [40.01-3,083.84], 35.77 [7.7-166.11], and 3.89 [1.35-11.21], respectively. On multivariate analysis, cardiac arrest at presentation and the TIMI risk score were found to be independent predictors of 14-day mortality with adjusted ORs of 136.49 [10.23-1821.27] and 2.67 [1.09-6.57], respectively.

**Table 2:** Univariate and multivariate logistic regression analysis for 14-day outcome CAD, coronary artery diseases; CI, confidence interval; OR, odds ratio; SBP, systolic blood pressure; TIMI, thrombolysis in myocardial infarction *significant

Characteristics	Univariate		Multivariate	
	OR [95% CI]	p-value	OR [95% CI]	p-value
Female	1.7 [0.61-4.72]	0.306	2.59 [0.43-15.58]	0.298
>65 years	2.06 [0.78-5.4]	0.143	1.03 [0.21-5.1]	0.972
Heart rate (per 5 bpm)	1.16 [1.01-1.33]	0.038*	0.94 [0.67-1.32]	0.739
Serum creatinine (ng/dL)	1 [0.94-1.07]	0.925	1.02 [0.94-1.11]	0.616
Troponin I (ng/dL)	1.01 [0.99-1.02]	0.427	1.01 [0.99-1.03]	0.531
Cardiac arrest	351.25 [40.01-3083.84]	<0.001*	136.49 [10.23-1,821.27]	<0.001*
Killip class II-IV	35.77 [7.7-166.11]	<0.001*	12.16 [0.19-772.82]	0.238
Hypertension	3.31 [0.43-25.52]	0.25	10.31 [0.03-3,505.28]	0.433
Diabetes	3.89 [1.35-11.21]	0.012*	2.17 [0.38-12.36]	0.382
Family history of CAD	1.68 [0.46-6.14]	0.433	5.6 [0.83-37.67]	0.076
Smoking	0.75 [0.24-2.34]	0.617	0.6 [0.06-5.91]	0.665
Obesity	1.28 [0.28-5.91]	0.747	7.27 [0.88-59.75]	0.065
Dyslipidemia	0.68 [0.15-3.04]	0.61	0.13 [0.01-1.7]	0.118
Sedentary lifestyle	1.12 [0.41-3.08]	0.825	0.6 [0.1-3.68]	0.585
TIMI score	3.32 [1.83-6.02]	<0.001*	2.67 [1.09-6.57]	0.032*

## Discussion

Patients with NSTEMI are likely to be of older age and present with multiple cardiovascular risk factors and are more prone to increased risk of cardiovascular complications. Hence, risk stratification modalities, such as the TIMI risk score, were found to be useful in stratification of high-risk patients. Hence, this study was conducted to assess the validity of the TIMI risk score for predicting 14-day mortality in NSTEMI patients in Pakistan. This study is the first of its kind in a population in Pakistan. We observed a reasonably acceptable prognostic discriminating ability of the TIMI risk score with an AUC of 0.788 (0.689-0.887), and the derived optimal cutoff of ≥4 had a moderate sensitivity of 77.78% (52.36%-93.59%) and a specificity of 68.09% (62.30%-73.49%), with 14-day mortality rates of 13.5% versus 2.0% (p < 0.001) for patients with TIMI scores of ≥4 and <4, respectively.

The study by Antman et al. [3] proposed the TIMI risk score for NSTEMI-ACS based on seven independent predictor variables. The proposed score and event rate after 14 days of revascularization (including recurrent MI, re-revascularization, and death) were found to have significant association with monotonically increasing event rate along the TIMI score in validation cohort. The AUC value of 0.788 in our study is consistent with the C-statistics of 0.74 reported for all-cause mortality in the parent study. The TIMI score was also validated using PRISM-PLUS database by Morrow et al. [14], with a similar observation of increasing event rate ranging from 7.7% at TIMI (1 or 2) to 30.5% at TIMI (6 or 7). Validation and subsequent adoption of this risk score in clinical routine can be particularly useful for a country like Pakistan, where every one in four middle-aged adult presents with coronary artery disease [15].

Our analysis also demonstrates that Killip class II-IV, increased heart rate, cardiac arrest upon presentation, and history of diabetes to be significantly associated with increased risk of 14-day mortality. With respect to univariate analysis, prior observations similarly indicate Killip class and diabetes to be associated with increased 14-day mortality [16]. Diabetes is one of the most prevalent non-communicable diseases in Pakistan, with up to 11.7% of the adult population living with it, thereby ensuring the importance of the TIMI risk score as a useful predictive tool in this population cohort [17]. In developing countries like Pakistan, our results may aid physicians in evaluating their patients using a cost-effective risk stratification tool; hence, TIMI can be used effectively for majority population in a publicly funded healthcare setup where limited funds are available [18].

Although maximum effort was undertaken to reduce bias, there are a few that should not be overlooked. First, our study was conducted in only one tertiary cardiac care center of Pakistan, which makes it difficult to generalize the results in other cardiac centers with different populations. To accurately evaluate the validity of the TIMI risk score, large sample size from several settings should be considered. Second, we did not take medications or prescribed therapeutic interventions into consideration, which may have influenced the prognosis of these patients. Further studies should be conducted to eliminate these limitations and provide further insight into the accuracy of TIMI risk score in patients with NSTEMI in Pakistan.

## Conclusions

The TIMI risk score is a useful and simple score for the stratification of patients with high risk of 14-day mortality with reasonably acceptable discriminating ability in patients with NSTEMI-ACS. Therefore, its use among clinicians is suggested to apply strategic and precise interventions in monitoring these patients. If used appropriately, this tool may prove to be a life-saving and cost-effective risk stratification tool in cardiac care settings.
